# Comparing Single and Dual Plating in Displaced Scapular Body Fractures: A Retrospective Study of Clinical and Functional Outcomes

**DOI:** 10.3390/jcm14134740

**Published:** 2025-07-04

**Authors:** Hsin-Hsin Lee, Hao-Chun Chuang, Wei-Chin Lin, Jou-Hua Wang, Ming-Hsien Hu, Pei-Yuan Lee, Hong-Lin Su, Chang-Han Chuang

**Affiliations:** 1 Department of Surgery, Taipei Veterans General Hospital, Taipei 112, Taiwan; 2 Department of Orthopaedic Surgery, Show Chwan Memorial Hospital, Changhua 500, Taiwan; lymphothebeast@gmail.com (W.-C.L.); minghsienhu@gmail.com (M.-H.H.); b1208b@ms26.hinet.net (P.-Y.L.); 3 Department of Orthopaedic Surgery, National Cheng Kung University Hospital, College of Medicine, National Cheng Kung University, Tainan 701, Taiwan; 4 Department of Orthopaedic Surgery, Kaohsiung Show Chwan Memorial Hospital, Kaohsiung 821, Taiwan; 5 Department of Post-Baccalaureate Medicine, College of Medicine, National Chung Hsing University, Taichung 402, Taiwan; suhonglin@nchu.edu.tw; 6 Department of Life Sciences, National Chung Hsing University, Taichung 402, Taiwan

**Keywords:** Scapula, Scapular body fracture, plate osteosynthesis, dual plating, functional outcome

## Abstract

**Background:** Scapular body fractures, when significantly displaced or malunited, can cause glenohumeral discomfort and functional disability. This study compares single- and dual-plating techniques in terms of pain, function, and active range of motion (aROM) in patients with scapular body fractures. **Methods:** Twenty-eight patients with scapular fractures were retrospectively analyzed, with sixteen undergoing single plating treatment and twelve dual plating treatment. The mean age was 44.9 years, and the mean follow-up was 14 months for single plating and 13.8 months for dual plating. Outcomes included Disabilities of the Arm, Shoulder and Hand (DASH) scores, the Visual Analog Scale (VAS) for pain, aROM measurements, and the time to return to work. Functional outcomes were assessed using two-way ANOVA with Šidák’s multiple comparisons test at 2 weeks, 4 weeks, 3 months, 6 months, and 1 year. The time to return to work was analyzed with survival analysis and a log-rank test. **Results:** The single plating group had higher DASH scores than the dual plating group at 2 weeks (44.88 ± 10.81 vs. 32.75 ± 6.05, *p* = 0.005), 4 weeks (28.50 ± 5.91 vs. 22.83 ± 4.24, *p* = 0.033), and 3 months (19.63 ± 2.45 vs. 16.00 ± 2.45, *p* = 0.004), indicating greater disability. VAS scores were also higher in the single plating group at 2 weeks (4.00 ± 1.21 vs. 2.33 ± 0.88, *p* = 0.002) and 4 weeks (2.50 ± 1.03 vs. 1.17 ± 0.94, *p* = 0.008), suggesting faster pain relief in the dual plating group. However, differences were no longer significant after 3 months. At 1 year, the dual plating group demonstrated better external rotation (73 ± 3° vs. 63 ± 5°, *p* = 0.032), with no significant differences in internal rotation, abduction, or forward flexion. Dual plating patients returned to work earlier (Hazard Ratio = 3.346, 95% CI: 1.208 to 9.269, *p* = 0.020). **Conclusions:** In the current cohort, dual plating for scapular fractures offers superior early pain relief and functional outcomes compared to single plating, along with better external rotation at 1 year and an earlier return to work. These findings suggest that dual plating may facilitate faster recovery and enhanced active range of motion in selected patients, a hypothesis that warrants further investigation through future randomized trials.

## 1. Introduction

Scapula fractures, accounting for 3% to 5% of all shoulder fractures, are not uncommon in clinical practice and may occasionally occur alongside clavicle and proximal humerus fractures [1,2]. Due to the scapula’s robust muscular envelope, which generally facilitates fracture healing, these injuries have traditionally been managed nonoperatively for decades [3,4,5]. Nevertheless, scapular malunions are not uncommon and can result in significant discomfort and functional impairments of the shoulder girdle [6,7]. These impairments often manifest as persistent pain, cosmetic deformities, impingement, and scapulothoracic dyskinesis [4,6,8,9].

To prevent scapular malunions, current surgical indications for scapular fractures include an intra-articular step-off exceeding 4 mm, double shoulder suspensory injuries, and significant scapular body deformities [10,11]. Specific criteria for surgical intervention in scapular body fractures have been identified, including medial/lateral displacement greater than 20 mm, shortening exceeding 25 mm, and angular deformity over 40° [10,11]. Despite surgical fixation, these fractures may still result in reduced external rotation range of motion and infraspinatus hypotrophy [10,12,13]. Among significantly displaced scapular body fractures the debate persists regarding the efficacy of single- versus dual-plate fixation. The traditional single plating at the lateral border offers advantages such as shorter operative time, reduced blood loss, and lower rates of implant prominence and removal [8,14]. In contrast, dual plating at both the lateral and medial borders has demonstrated increased fixation strength, favorable radiographic outcomes, low malunion rates, and minimal complications [11,15,16].

Existing evidence suggests that single plating offers advantages such as shorter operative time and reduced blood loss, whereas dual plating provides superior biomechanical stability. However, to the best of our knowledge, no prior studies have directly compared the functional outcomes, radiographic results, malunion rates, or time to return to work between single- and dual-plating techniques for significantly displaced scapular body fractures. To address this clinical uncertainty, we conducted a retrospective analysis at a tertiary trauma center, comparing single- and dual-plate fixation for scapular body fractures involving both borders. We hypothesized that dual plating enhances the functional recovery and range of motion without increasing complications after a minimum follow-up period of one year.

## 2. Materials and Methods

### 2.1. Population

A total of 28 cases of scapular body fractures, classified as OTA-14B fixation according to the AO-OTA (Arbeitsgemeinschaft für Osteosynthesefragen—Orthopaedic Trauma Association) classification system, treated at a single trauma center between 2014 and 2019, were included in this study [17]. We included only cases from 2014 to 2019 to ensure both surgical consistency and adequate follow-up duration. Indications for surgical intervention were based on established criteria and included medial/lateral displacement greater than 10 mm (reduced to 5 mm for cases with double disruptions and 10 mm when combined with a 30-degree angulation), shortening exceeding 25 mm, a glenopolar (GP) angle equal to or less than 22 degrees, and body angulation equal to or greater than 40 degrees [10,11,18].

All surgical procedures were performed by an experienced trauma surgeon. The therapeutic modalities of single plating and dual plating, along with their associated risks and benefits, were thoroughly discussed with patients using a shared decision-making approach. Patients opting for dual plating were informed of potential risks, including increased blood loss, prolonged operative time, and implant prominence. Fixation devices included anatomical lateral border locking plates and anatomical medial border locking plates (Acumed; Hillsboro, OR, USA).

The institutional review board of the Show Chwan Memorial Hospital reviewed and approved the study protocol (SCMH_IRB_1100712). All the participants provided written informed consent.

### 2.2. Surgical Technique

**Figure 1:**
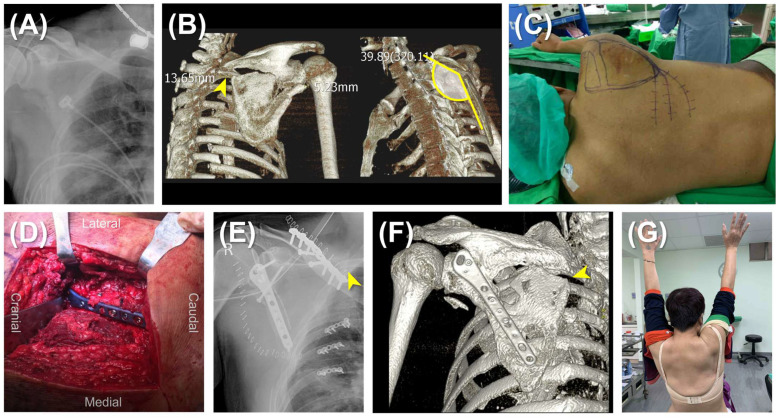
Single lateral plating for scapular fracture. (**A**) Preoperative imaging of a 58-year-old woman with a right scapular fracture (AO/OTA 14B(lm)) accompanied by ipsilateral clavicle and multiple rib fractures, hemothorax, and pneumothorax. (**B**) CT reconstruction demonstrating a marked displacement and 40° angulation of the scapular body at its lateral and medial borders (yellow arrowhead). (**C**) Patient positioned in the left lateral decubitus position for surgical stabilization. (**D**) Intraoperative view via the mirror Judet approach showing fixation of the scapular fracture with a single lateral anatomical locking plate and concurrent reduction and stabilization of multiple rib fractures. (**E**) Postoperative imaging revealing a persistent medial gap and resulting malunion (yellow arrowhead). (**F**) Another case of scapular body fracture. At 12-month follow-up, CT confirms solid osteosynthesis of the lateral border but malunion of the medial border persists (yellow arrowhead). (**G**) Clinical photograph of the 58-year-old woman at 12 months demonstrating restoration of full active shoulder abduction.

The surgery was carried out with the patients under general anesthesia and positioned in lateral decubitus. In the single plating group, a lateral straight incision was made and the modified Judet approach was generally adopted. The mirror Judet approach was utilized in cases with concomitant rib fractures requiring fixation [19]. The lateral border of the scapula was exposed between the teres minor and infraspinatus muscles, ensuring visualization and protection of the suprascapular nerve and vessels. The lateral border was reduced under direct visualization and fluoroscopic assistance. Fixation was achieved using an anatomical locking plate (Acumed, OR, USA) (Figure 1).

**Figure 2:**
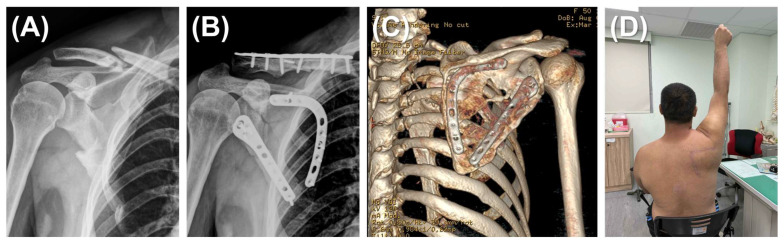
Dual plating for scapular fracture. (**A**) A 52-year-old man presented with a left scapular fracture (AO/OTA 14B type) and an ipsilateral clavicle fracture. The scapular body exhibited comminution along both the medial and lateral borders. (**B**) Through a modified Judet approach, the scapular fracture was reduced and stabilized using dual plating (lateral and medial borders). The clavicle fracture was addressed with a standard fixation procedure. (**C**) A 3D reconstruction at the 16-month postoperative follow-up, after removal of the clavicular implants, demonstrating successful osteosynthesis of the scapular body fracture without malalignment. (**D**) Clinical photograph of the 52-year-old man at 12 months demonstrating restoration of full active shoulder abduction.

In the dual plating group, two incisions were made simultaneously: a reverse L-shaped incision and a lateral straight incision. The reverse L-shaped incision started from the scapular spine, angled sharply at the superomedial angle of the scapula, and continued along the medial border to the inferior angle. The medial border was reduced first and stabilized with a scapula medial anatomical locking plate (Acumed; OR, USA) to facilitate reduction and the stabilization of fracture fragments. Once the medial plate was securely positioned, the lateral border was reduced and fixed using the same procedure described for single plating (Figure 2).

To complete the procedure, the glenoid fossa was meticulously evaluated with fluoroscopy, and the shoulder was carefully manipulated to avoid intra-articular screw placement, ensuring joint integrity. Postoperatively, the patient’s shoulder was immobilized with an arm sling for a minimum of one week to promote initial healing and prevent undue stress on the surgical site.

### 2.3. Postoperative Rehabilitation

Passive-assisted range of motion exercises were initiated one week after the operation under the guidance and supervision of a skilled physical therapist. These exercises aimed to gently mobilize the joint and prevent stiffness while minimizing the risk of compromising the surgical repair. At two weeks postoperation, active-assisted range of motion exercises were introduced, allowing the patient to actively participate in the movements with some assistance, gradually increasing the demand on the healing tissues. After three weeks the patient progressed to active range of motion exercises, where they performed the movements independently, further enhancing strength and function. Full range of motion exercises were initiated six weeks postoperation, enabling the patient to engage in a complete range of motion without restrictions, promoting optimal recovery and functional outcomes.

### 2.4. Follow-Up

Patients were included only if they completed a minimum follow-up of 1 year, had complete radiographic examinations and physical evaluations at 1 year postoperatively, and filled out functional outcome assessment sheets without missing data at five time points. Postoperative radiographic examinations included scapular Grashey view, scapular Y view, and computed tomography. Malunion was defined as body angulation exceeding 10 degrees, displacement over 5 mm, and a difference in glenoid polar angle over 10 degrees compared with the contralateral side [6]. The same physical therapist assessed postoperative shoulder range of motion using a goniometer and compared the results with the contralateral side at 1 year postoperatively. Assessed ROM included forward flexion, scapular-plane abduction, external rotation, and internal rotation (at thoracic level). Patient-reported outcomes were measured using the Disabilities of the Arm, Shoulder, and Hand (DASH) questionnaire and the Visual Analog Scale (VAS) at 2 weeks, 4 weeks, 3 months, 6 months, and 12 months postoperatively. The status of if they were working and the time to return to work were also collected.

### 2.5. Statistical Analysis

Sample size estimation was based on prior studies, with an effect size of 1 from a previous study evaluating patients with shoulder disorders using the DASH score [20]. With 80% power, α = 0.05, and one-tail examination, the minimum required sample size was determined to be approximately 14 in each group.

Continuous data were presented as mean ± standard deviation, while categorical data were presented as the number of incidences and the percentage. The ROM at one year was compared using Student’s t-test, and the complications were compared using Chi-square analysis. The time-to-return-to-work data were analyzed using Kaplan–Meier survival estimates, and differences between the groups were assessed with the log-rank test. The DASH score and VAS score were compared using a two-way repeated measures ANOVA with Šídák’s multiple comparisons test at the five time points. A *p*-value < 0.05 was considered statistically significant. Analyses were conducted using SPSS Statistics, version 17.0 (IBM Corporation, Armonk, NY, USA).

## 3. Results

### 3.1. Demographics

A total of 28 patients completed the one-year follow-up and comprehensive examinations, including 16 treated with single plating and 12 with dual plating. The mean age was 43.3 ± 9.1 years in the single plating group and 47.1 ± 12.5 years in the dual plating group. Concurrent rib fracture fixation was performed in two patients from the single plating group and one patient from the dual plating group. The mean Injury Severity Score (ISS) was 25.7 ± 13.3 in the single plating group and 27.2 ± 14.5 in the dual plating group (Table 1).

For all 28 patients the combined surgical time was 96.9 ± 25.1 min, blood loss was 244.7 ± 97.9 mL, transfusion volume was 437.4 ± 250.2 mL, and the length of hospitalization was 9.1 ± 3.7 days. The dual plating group exhibited significantly longer surgical times and greater blood loss compared to the single plating group. The surgical time in the dual plating group was, on average, 23 min longer (*p* = 0.010), while blood loss was 88 mL greater (*p* = 0.014).

### 3.2. Patient Reported Outcome Measurements

**Figure 3:**
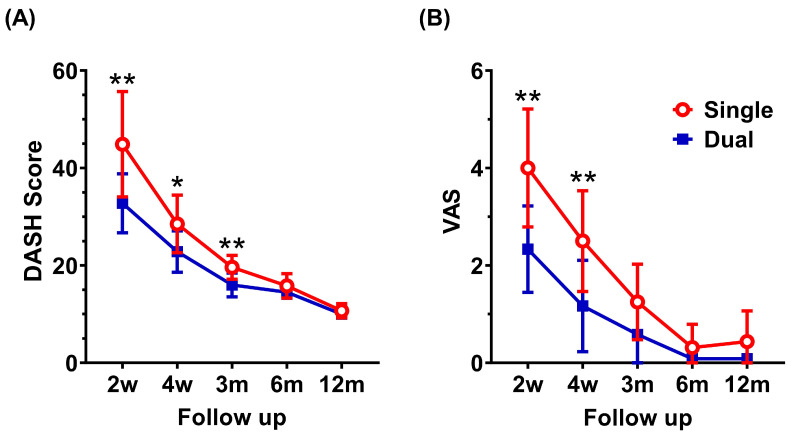
Patient-reported outcome measurements. (**A**) During the first three postoperative months, the DASH scores were significantly higher in the single plating group compared to the dual plating group, reflecting greater disability among patients treated with single plating. The difference between groups diminished over time and was no longer statistically significant beyond 6 months. (**B**) Within the first four postoperative weeks, VAS scores were significantly higher in the single plating group compared to the dual plating group, indicating higher pain levels in the single plating group. At subsequent time points, the differences in VAS scores between the groups were not statistically significant. A two-way ANOVA with post hoc Šídák’s multiple comparisons test was used to analyze the simple main effects of the number of plates on patient-reported outcome measurements at different follow-up time points. *, *p* < 0.05; **, *p* < 0.01.

A two-way ANOVA revealed a statistically significant interaction between the number of plates and time on the DASH score (F(4, 104) = 9.00, *p* < 0.001). Šídák’s multiple comparisons test was used to examine the simple main effects of the number of plates at different follow-up time points. Within the first 3 postoperative months, the DASH score was significantly higher in the single plating group compared to the dual plating group, indicating greater disability among patients undergoing single plating treatment. The mean differences (±standard deviations) were 12.13 ± 4.57 at 2 weeks, 5.67 ± 2.71 at 4 weeks, and 3.63 ± 1.33 at 3 months postoperative (*p* = 0.005, 0.033, and 0.004, respectively). However, the differences in DASH scores at 6 months (1.31 ± 1.03) and 12 months (0.69 ± 0.45) postoperative were not statistically significant (Figure 3A).

Similarly, another two-way ANOVA revealed a statistically significant interaction between the number of plates and time on the VAS score (F(4, 104) = 7.62, *p* < 0.001). Šídák’s multiple comparisons test was used to analyze the simple main effects of the number of plates at various follow-up time points. Within the first 4 postoperative weeks, the VAS score was significantly higher in the single plating group compared to the dual plating group, suggesting that patients undergoing single plating experienced more pain. The mean differences (± standard deviations) were 1.67 ± 0.56 at 2 weeks and 1.33 ± 0.53 at 4 weeks postoperative (*p* = 0.001 and 0.008, respectively). In contrast, the differences in VAS scores at 3 months (0.67 ± 0.39), 6 months (0.23 ± 0.21), and 12 months (0.35 ± 0.25) postoperative did not reach statistical significance (Figure 3B).

These analyses indicated that patients who underwent single plating experienced greater disability and more pain during the first postoperative month compared to those who received dual plating.

### 3.3. Return to Work Status

**Figure 4:**
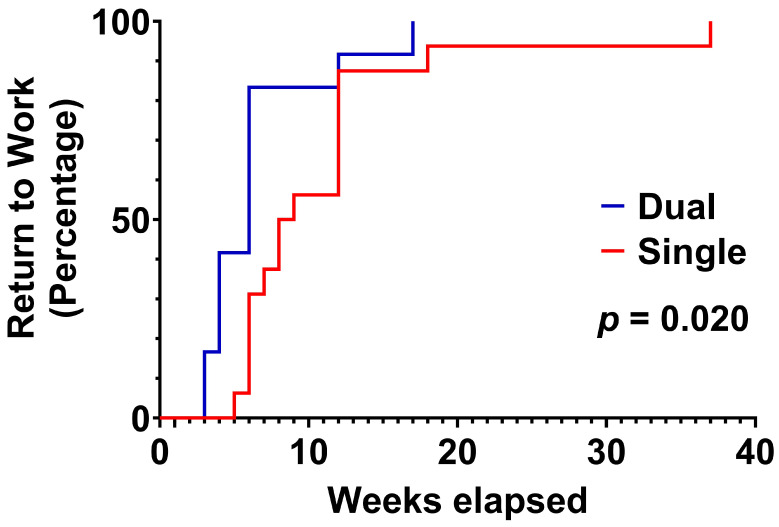
Time to return to work. Patients in the dual plating group returned to work significantly earlier than those in the single plating group (χ^2^ = 5.395, df = 1, *p* = 0.020, log-rank [Mantel–Cox] test). The hazard ratio, calculated using the Mantel–Haenszel method, indicated that patients in the dual plating group were over three times more likely to return to work earlier than those in the single plating group (HR = 3.346, 95% CI: 1.208 to 9.269).

All patients successfully returned to work. The median time to return to work was 8.5 weeks in the single plating group and 6 weeks in the dual plating group (Figure 4). Survival analysis demonstrated that patients in the dual plating group returned to work significantly earlier than their single plating counterparts, as evidenced by both the log-rank (Mantel–Cox) test (χ^2^ = 5.395, df = 1, *p* = 0.020) and the Gehan–Breslow–Wilcoxon test (χ^2^ = 7.623, df = 1, *p* = 0.006). These results indicate significant differences in return-to-work times between the groups. The hazard ratio from the Mantel–Haenszel method was 3.346 (95% CI: 1.208 to 9.269), indicating that patients in the dual plating group were over three times more likely to return to work earlier than those in the single plating group.

### 3.4. Range of Motion

Regarding range of motion, the dual plating group demonstrated a significant advantage over the single plating group in external rotation at the 12-month postoperative follow-up. The average external rotation was 63 ± 5° in the single plating group and 73 ± 3° in the dual plating group (*p* = 0.032).

**Figure 5:**
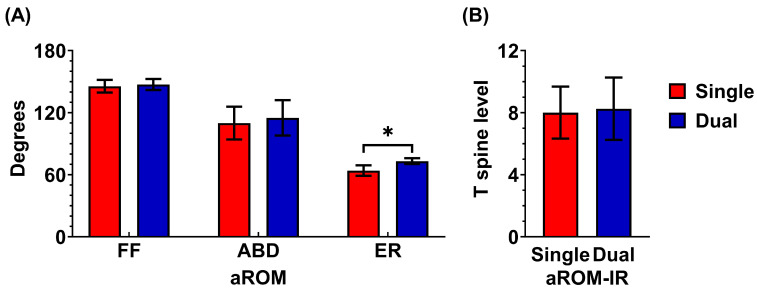
Range of motion at one-year postoperative. (**A**) At the 12-month postoperative follow-up, the dual plating group demonstrated a significant advantage over the single plating group in external rotation (73 ± 3° vs. 63 ± 5°, *p* = 0.032, Student’s *t*-test). However, no significant differences were observed between the two groups in abduction, or forward flexion. (**B**) No significant difference was observed between the two groups in internal rotation, either. *, *p* < 0.05.

There were no significant differences between the two groups in internal rotation, abduction, or forward flexion at the 12-month follow-up. In the single plating group, the average forward flexion was 146 ± 6°, abduction was 110 ± 16°, external rotation was 63 ± 5°, and internal rotation reached the T8 spine level. In comparison, the dual plating group had an average forward flexion of 147 ± 5°, abduction of 115 ± 17°, external rotation of 73 ± 3°, and internal rotation also at the T8 spine level (Figure 5).

### 3.5. Complications

In terms of complications, the single plating group experienced three cases of malunion (3/16), whereas no cases of malunion were observed in the dual plating group (0/12) (*p* = 0.239, Fisher’s exact test). The malunion cases in the single plating group included two instances of scapular body angulation exceeding 10 degrees and one case of a glenoid polar angle decrease of 10 degrees or more compared to the contralateral side. No wound complications requiring debridement or skin flap procedures were observed in either group.

### 3.6. Post Hoc Power Analysis

Although our groups comprised 16 and 12 patients rather than the planned 14 per arm, we performed post hoc power analyses on our co-primary endpoints (DASH and VAS scores). With a within-subject correlation of 0.5 for DASH and 0.4 for VAS, the achieved power was 0.74 for DASH and 0.90 for VAS. These results indicate that our study remains adequately powered—particularly for detecting differences in pain (VAS)—and that our findings are robust despite the slight group-size imbalance.

## 4. Discussion

The literature supports the facilitation of functional recovery following lateral plating for severely displaced scapular fractures but has not extensively explored the benefits of dual plating compared with single lateral plating [21]. This comparative study underscores the advantages of dual plating over single plating for scapular fractures, particularly in accelerating functional recovery, enabling an earlier return to work, and preserving external rotational range of motion. These findings suggest that dual plating is a valuable surgical option, especially for patients prioritizing a faster recovery and an improved range of motion.

The management of extra-articular scapular fractures has evolved significantly over recent decades [22]. Studies have demonstrated poor outcomes with the nonoperative treatment of certain fracture patterns, particularly those with a glenopolar angle of <20° (normal glenopolar angle = 35–40°) [22,23,24,25,26]. In a classic cohort of conservatively treated scapular fractures, Nordqvist reported that up to half of patients experienced shoulder symptoms, including pain, functional deficits, and reduced motion or strength [27]. Recent reviews corroborate these findings, highlighting poor outcomes of conservative treatment for severely displaced scapular fractures. For example, Sernandez reported that nonoperative glenoid fractures yield the lowest functional scores, with malunion, the need for additional surgeries, and post-traumatic arthritis being the most common complications [22]. With the advent of anatomical locking plate systems, recent studies on operatively treated scapular fractures have shown expedited functional recoveries, though with some limitation in the external rotational range of motion [28,29,30]. However, prior studies primarily focused on single lateral border plating or combined single and dual plating without distinction in their analyses [11,28]. This study directly compares dual plating with single lateral plating. The findings emphasize that dual plating enhances fracture reduction, particularly by minimizing medial border malunion. Furthermore, the results demonstrate that dual plating facilitates functional recovery and improves external rotational range of motion. In summary, this study aligns with recent reports advocating for the surgical fixation of severely displaced scapular fractures and adds evidence that, in high-demand patients or those seeking rapid functional recovery, dual plating is a safe and effective alternative.

Reduced external rotation is a common sequela following scapular fractures. The mean external rotational ROM reported by Herrera et al. and Schroder et al. was 61° and 66°, respectively [11,30]. Some studies suggest that external rotational ROM deficits may stem from the surgical approach [31]. For instance, the classic Judet approach, often employed for complex scapular fracture reductions and cases with central floating fragments, requires extended exposure for surgical fixation using a buttress plate [13]. In contrast, Fandridis et al. proposed a posterior subdeltoid, external rotator-preserving approach for the reduction and fixation of displaced extra-articular scapular fractures [31]. Interestingly, in our study, the dual plating group—requiring dual surgical approaches—achieved better external rotational ROM (73°) compared to the single plating group (63°). We hypothesize that this improvement is related to the restoration of rotator cuff muscle insertions following reduction in the fractured medial scapular border. Sung et al. reported that lower trapezius/upper trapezius muscle activity ratios and serratus anterior/upper trapezius co-contraction indices increase as body posture shifts away from a sitting position [32]. We deduced that restoring the glenopolar angle and medial scapular border facilitates reattachment of the infraspinatus and teres minor muscles to their original footprints, thereby restoring their function in externally rotating the glenohumeral joint.

Surgical fixation of scapular fractures facilitates early functional recovery, but the complex anatomy of the scapula poses challenges in selecting appropriate implants [3]. Asihin et al. advised that fixation of the lateral and medial borders of the scapular body requires low-profile plates with short screw lengths ranging from 6 to 10 mm. When anatomical scapular plates are unavailable, they recommended using a combination of 2.7 mm mini plates, 3.5 mm reconstruction plates, and 1/3 tubular plates [13]. In this study, scapular body anatomical plates were employed. Evidence supports the use of these newer scapular-specific locking plate systems, highlighting their benefits, including the absence of postoperative complications such as infection, non-union, or internal fixation failure [28,29,30].

Surgical management of scapular body fractures has increasingly embraced the Brodsky approach—a posterior, subdeltoid exposure that spares the rotator interval and infraspinatus—because it minimizes soft-tissue trauma and has been shown to preserve external-rotation strength and accelerate early functional recovery [31]. However, by focusing on the lateral column, this technique provides less access to the medial scapular border and ventral surface, which can hinder fixation of highly comminuted or bicolumnar fractures [31,33]. For these reasons, our current study employs the Judet approach, which is a more extensile posterior exposure that mobilizes the infraspinatus to afford comprehensive visualization of both lateral and medial columns, facilitating secure fixation of complex fracture patterns and offering surgeons familiar anatomical landmarks [33]. This well-established technique, however, carries its own trade-offs, including a longer skin incision and greater muscle mobilization that may increase postoperative wound morbidity and risk of infraspinatus atrophy, thereby potentially prolonging rehabilitation compared with the less invasive Brodsky exposure [34].

This study has several limitations that warrant acknowledgment. First, the relatively small sample size may limit the generalizability of the findings and reduce the statistical power to detect subtle differences. Second, the cohort was not stratified by occupational demands, which may influence return-to-work outcomes, as the physical intensity of a patient’s job can significantly affect recovery and the timing of return. Third, the retrospective study design introduces inherent biases, and future prospective or randomized trials are needed to validate these results. Fourth, the study focused exclusively on extra-articular scapular body fractures, and the recommendations should be interpreted with caution for cases involving the articular surface of the glenoid. Finally, the diversity of surgical approaches and the wide range of available implants further constrain the generalizability of the results to broader clinical contexts.

## 5. Conclusions

This comparative study underscores the advantages of dual plating over single plating for scapular fractures, particularly in accelerating functional recovery, enabling an earlier return to work, and preserving external rotational range of motion. These findings suggest that dual plating may facilitate faster recovery and enhanced active range of motion in selected patients, a hypothesis that warrants further investigation through future randomized trials.

## Figures and Tables

**Table 1:** Demographic and fracture classification.

Characteristics	All	Single Plating	Dual Plating	*p*-Value
Case numbers	28	16	12	
Age (years)	44.9 ± 10.9	43.3 ± 9.1	47.1 ± 12.5	0.384
Sex				0.907
Male	19	11	8	
Female	9	5	4	
Rib fixation	3	2	1	
Injury severity score	26.3 ± 13.9	25.7 ± 13.3	27.2 ± 14.5	0.782
Surgical time (minutes)	97 ± 25	87 ± 25	110 ± 19	0.010
Blood loss (ml)	245 ± 98	207 ± 93	295 ± 83	0.014
Transfusion (ml)	437 ± 250	407 ± 282	478 ± 205	0.447

Data were presented as mean ± standard deviation.

## Data Availability

The original contributions presented in this study are included in the article. Further inquiries can be directed to the corresponding author.

## References

[1] Veysi V.T., Mittal R., Agarwal S., Dosani A., Giannoudis P.V. (2003). Multiple trauma and scapula fractures: So what?. J. Trauma.

[2] Rowe C.R. (1963). Fractures of the Scapula. Surg. Clin. N. Am..

[3] Gosens T., Speigner B., Minekus J. (2009). Fracture of the scapular body: Functional outcome after conservative treatment. J. Shoulder Elb. Surg..

[4] Ada J.R., Miller M.E. (1991). Scapular fractures. Analysis of 113 cases. Clin. Orthop. Relat. Res..

[5] Jones C.B., Sietsema D.L. (2011). Analysis of operative versus nonoperative treatment of displaced scapular fractures. Clin. Orthop. Relat. Res..

[6] Cole P.A., Talbot M., Schroder L.K., Anavian J. (2011). Extra-articular malunions of the scapula: A comparison of functional outcome before and after reconstruction. J. Orthop. Trauma.

[7] Gauger E.M., Cole P.A. (2011). Surgical technique: A minimally invasive approach to scapula neck and body fractures. Clin. Orthop. Relat. Res..

[8] Bartoníček J., Frič V. (2011). Scapular body fractures: Results of operative treatment. Int. Orthop..

[9] Bartonícek J., Cronier P. (2010). History of the treatment of scapula fractures. Arch. Orthop. Trauma Surg..

[10] Vidović D., Benčić I., Ćuti T., Bakota B., Bekić M., Dobrić I., Sabalić S., Blažević D. (2021). Surgical treatment of scapular fractures: Results and complications. Injury.

[11] Cole P.A., Gauger E.M., Herrera D.A., Anavian J., Tarkin I.S. (2012). Radiographic follow-up of 84 operatively treated scapula neck and body fractures. Injury.

[12] Porcellini G., Palladini P., Congia S., Palmas A., Merolla G., Capone A. (2018). Functional outcomes and clinical strength assessment after infraspinatus-sparing surgical approach to scapular fracture: Does it really make a difference?. J. Orthop. Traumatol..

[13] Mohd Asihin M.A., Bajuri M.Y., Ganaisan P.K., Ahmad A.R. (2019). Open Reduction and Internal Fixation of Extraarticular Scapular Neck and Body Fractures with Good Short Term Functional Outcome. Front. Surg..

[14] Ao R., Yu B., Zhu Y., Jiang X., Shi J., Zhou J. (2018). Single lateral versus medial and lateral plates for treating displaced scapular body fractures: A retrospective comparative study. J. Shoulder Elb. Surg..

[15] Katthagen J.C., Sußiek J., Frank A., Wermers J., Schliemann B., Raschke M.J. (2022). Double plating is associated with higher fixation strength than single plating in osteoporotic fractures of the scapular spine: A biomechanical study. Arch. Orthop. Trauma Surg..

[16] Gao M., Nie D., Chang Y., Xie W., Wang Y., Pu X., Zhang W., Luo W. (2019). Internal fixation of lateral and medial borders for displaced scapular body fractures via minimally invasive approach: Results of 23 cases. Zhejiang Da Xue Xue Bao Yi Xue Ban.

[17] Audigé L., Kellam J.F., Lambert S., Madsen J.E., Babst R., Andermahr J., Li W., Jaeger M. (2014). The AO Foundation and Orthopaedic Trauma Association (AO/OTA) scapula fracture classification system: Focus on body involvement. J. Shoulder Elb. Surg..

[18] Cole P.A., Gauger E.M., Schroder L.K. (2012). Management of scapular fractures. J. Am. Acad. Orthop. Surg..

[19] Chuang C.-H., Huang C.-K., Li C.-Y., Hu M.-H., Lee P.-Y., Wu P.-T. (2022). Surgical stabilization of the ipsilateral scapula and rib fractures using the mirror Judet approach: A preliminary result. BMC Musculoskelet. Disord..

[20] Schmitt J.S., Di Fabio R.P. (2004). Reliable change and minimum important difference (MID) proportions facilitated group responsiveness comparisons using individual threshold criteria. J. Clin. Epidemiol..

[21] Hu Y., Shi H., Wang F., Ren G., Cheng R., Zhang Z. (2019). Functional outcomes of extra-articular scapula fracture fixation with distal humeral Y-type locking plate: A retrospective study. J. Orthop. Surg. Res..

[22] Sernandez H.C., Riehl J.T., Fogel J. (2024). Sling and forget it? A systematic review of operative versus nonoperative outcomes for scapula fractures. J. Shoulder Elb. Surg..

[23] Zlowodzki M., Bhandari M., Zelle B.A., Kregor P.J., Cole P.A. (2006). Treatment of scapula fractures: Systematic review of 520 fractures in 22 case series. J. Orthop. Trauma.

[24] Romero J., Schai P., Imhoff A.B. (2001). Scapular neck fracture--the influence of permanent malalignment of the glenoid neck on clinical outcome. Arch. Orthop. Trauma Surg..

[25] Bozkurt M., Can F., Kirdemir V., Erden Z., Demirkale I., Başbozkurt M. (2005). Conservative treatment of scapular neck fracture: The effect of stability and glenopolar angle on clinical outcome. Injury.

[26] van Noort A., van Kampen A. (2005). Fractures of the scapula surgical neck: Outcome after conservative treatment in 13 cases. Arch. Orthop. Trauma Surg..

[27] Nordqvist A., Petersson C. (1992). Fracture of the body, neck, or spine of the scapula. A long-term follow-up study. Clin. Orthop. Relat. Res..

[28] Schroder L.K., Gauger E.M., Gilbertson J.A., Cole P.A. (2016). Functional Outcomes After Operative Management of Extra-Articular Glenoid Neck and Scapular Body Fractures. J. Bone Jt. Surg. Am..

[29] Cole P.A., Gilbertson J.A., Cole P.A. (2017). Functional Outcomes of Operative Management of Scapula Fractures in a Geriatric Cohort. J. Orthop. Trauma.

[30] Herrera D.A., Anavian J., Tarkin I.S., Armitage B.A., Schroder L.K., Cole P.A. (2009). Delayed operative management of fractures of the scapula. J. Bone Jt. Surg. Br..

[31] Fandridis E., Anastasopoulos P.P., Alexiadis G., Nomikarios D., Spyridonos S., Hertel R. (2018). Posterior subdeltoid and external rotators preserving approach for reduction and fixation of displaced extra-articular fractures of the scapula. Eur. J. Orthop. Surg. Traumatol..

[32] Sung J.-H., Jung W., Wang J., Kim J.-H. (2023). The Effects of Body Positions and Abduction Angles on Shoulder Muscle Activity Patterns during External Rotation Exercises. Healthcare.

[33] Harmer L.S., Phelps K.D., Crickard C.V., Sample K.M., Andrews E.B., Hamid N., Hsu J.R. (2016). A Comparison of Exposure Between the Classic and Modified Judet Approaches to the Scapula. J. Orthop. Trauma.

[34] Zhang P., Song X., Li P., He S., Yang Z., Xu F., Xu X., Lu J., Cao C., Zhou L. (2024). Minimally Invasive Combined Medial and Lateral Approach for Treating Displaced Scapular Body and Neck Fractures. Med. Sci. Monit..

